# Nosocomial Coronavirus Disease Outbreak Containment, Hanoi, Vietnam, March–April 2020

**DOI:** 10.3201/eid2701.202656

**Published:** 2021-01

**Authors:** Cuong Duy, Vuong Minh Nong, An Van Ngo, Tra Thu Doan, Tuan Quang Nguyen, Phuong Thai Truong, Linus Olson, Mattias Larsson

**Affiliations:** Bach Mai Hospital, Hanoi, Vietnam (C.D. Do, V.M. Nong, A.V. Ngo, T.T. Doan, T.Q. Nguyen, P.T. Truong);; Karolinska Institutet, Stockholm, Sweden (L. Olson, M. Larsson)

**Keywords:** COVID-19, Vietnam, quarantine, contact tracing, testing, outbreak, pandemic, respiratory infections, severe acute respiratory syndrome coronavirus 2, SARS-CoV-2, SARS, coronavirus disease, zoonoses, viruses, coronavirus

## Abstract

We report on the public health response generated by an outbreak of coronavirus disease (COVID-19) that occurred during March 2020 at Bach Mai Hospital (BMH) in Hanoi, northern Vietnam’s largest hospital complex. On March 18, a total of 3 distinct clusters of COVID-19 cases were identified at BMH. Diagnosis of the initial 3 COVID-19 cases led to contact tracing, symptom screening, and testing of 495 persons and limited quarantine of affected institutes or departments. When 27 staff members in the catering company tested positive for SARS-CoV-2, the entire BMH staff (7,664 persons) was put under quarantine. Contact tracing in the community resulted in an additional 52,239 persons being quarantined. After 3 weeks, the hospital outbreak was contained; no further spread occurred in the hospital. Rapid screening of cases, extensive testing, prompt quarantine, contact tracing, and social distancing contributed to prevent community transmission in Hanoi and northern Vietnam.

In Vietnam, as of September 19, 2020, there were 1,068 laboratory-defined cases of the coronavirus disease (COVID-19) and 35 deaths. The outbreak in Vietnam consisted of 2 waves: the first was during January 22–July 24 with imported cases from countries in the Asia–Pacific region and Europe (*1*–*3*), resulting in 417 cases and no deaths; the second wave began on July 25 in Da Nang, central Vietnam, with community transmission, resulting in 551 cases and 35 deaths (*4*).

Vietnam, a middle-income country in Southeast Asia with a population of »100 million and a long, porous border with China, had relatively few cases of COVID-19 and no deaths during the first wave of the outbreak. When the epidemic in China was first acknowledged in late December 2019, the government of Vietnam implemented rapid response and containment by investigation, contact tracing, and quarantine as well as broader community mitigation measures with substantial nonpharmacologic interventions (*5*). The government first strengthened border control measures on January 3; body temperature screening and health declarations by persons entering Vietnam were implemented on January 22. After a case of COVID-19 was detected in Vietnam on January 22 (*6*), the border with China was closed, and all persons entering Vietnam were placed in 14 days’ quarantine at centralized facilities. Persons who were suspected of being infected and who had a travel history to Wuhan or Hubei Province in China before January 1, as well as their direct contacts, were also traced and placed in quarantine. Steering committees for COVID-19 prevention were established at each administrative division level, from province to district and commune, under the overall direction of a national committee headed by a deputy prime minister. Tracing was performed by local Center for Disease Control health workers and police forces using flight data and residence information. In addition, a health declaration system was developed on both web and mobile platforms for persons to report their symptoms and suspected cases in nearby living areas. The communication strategies were prepared in early January from various channels, including national and local TV programs, official press, and social media (*5*,*7*). All schools and universities remained closed after Tet (the lunar new year holiday) during January 23–May 4. At centralized facilities, quarantined persons were tested for severe acute respiratory syndrome coronavirus 2 (SARS-CoV-2) >2 times. 

In January, the first 6 positive cases in Vietnam were diagnosed by the whole-genome sequencing method, with an average of 3–4 days for returning the results. Nasopharyngeal samples were collected at the quarantine sites, then transferred to reference laboratories. Four national institutes act as reference laboratories for different regions of the country. Three of the 4 reference laboratories diagnosed the first 6 cases, including National Institute of Hygiene and Epidemiology (NIHE) in Hanoi, Pasteur Institute in Ho Chi Minh City, and Pasteur Institute in Nha Trang. From January 31 onward, the real-time reverse transcription PCR (RT-PCR) method was widely applied, which helped reduce the time for laboratory confirmation to 6 hours. The test kits were first donated by the World Health Organization (WHO), then provided by Viet-A Corporation (https://www.vietacorp.com). 

During January 22–February 13, a total of 16 cases were detected in Vietnam during the first COVID-19 epidemic phase. Among these were 3 cases that were imported from Hubei Province in China to Vinh Phuc, a province near Hanoi; these 3 patients transmitted COVID-19 to 8 other persons, among them a 3-month-old infant (*8*). In response, an entire commune of 10,600 persons was placed in lockdown for 3 weeks. This early response and containment strategy was effective in preventing community transmission during the first phase of the pandemic, and all 16 patients have fully recovered from their illnesses (*9*).

Nosocomial transmission of SARS-CoV-2 has the potential to spark community transmission. In Italy, for example, the national outbreak was initiated by nosocomial transmission of SARS-CoV-2 from a patient in hospital in Codogno, Lombardy, whose delayed diagnosis (36 hours after admission) led to infection of many healthcare workers and other inpatients (*10*). Globally, healthcare workers were overrepresented among COVID-19 cases because they had a high level of exposure, especially those working in triage and COVID-19 screening and testing, along with healthcare workers who had direct patient contact in infectious disease and intensive care departments. In China, healthcare workers had 3.8% of all COVID-19 cases, and 14.8% of them had severe or critical illnesses (*11*).

On March 18 and March 19, the first 2 COVID-19 cases in healthcare workers at Bach Mai Hospital (BMH) in Hanoi were reported, leading to a widespread investigation and response effort at the hospital. We describe how a nosocomial COVID-19 outbreak in one of the largest hospitals in Vietnam was contained through rigorous testing, active case finding, contact tracing, and whole-hospital quarantine.

## Methods

### Setting

**Figure 1:**
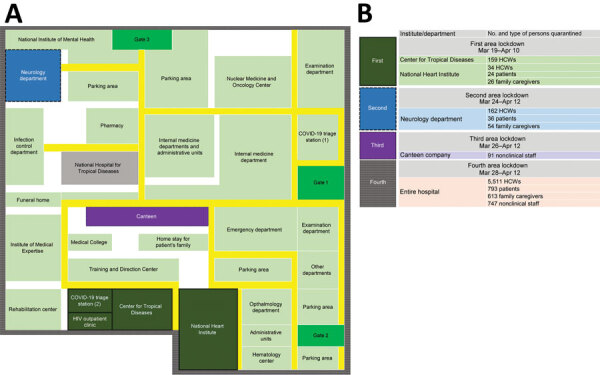
Hospital floor plan and timeline of lockdowns during outbreak of severe acute respiratory syndrome coronavirus 2 infections at Bach Mai Hospital complex in Hanoi, Vietnam. A) Hospital floor plan. B) Details of departmental or institution lockdowns. COVID-19, 2019 coronavirus disease; HCW, healthcare worker.

BMH is Hanoi’s largest national tertiary general hospital, with nearly 3,000 inpatient beds and an average of 5,000 outpatients per day. The hospital has 34 clinical centers, institutes, and departments and 6 paraclinical departments, with >6,000 healthcare workers and nonclinical staff. Three affiliated national institutes are under BMH management: National Heart Institute (NHI), National Institute of Mental Health, and National Institute of Medical Expertise. The first branch of the National Hospital for Tropical Diseases (NHTD) is also located inside the BMH area, but has been a freestanding hospital since 2006. The NHTD has a second branch that was the designated hospital for COVID-19 patients in northern Vietnam, located in a suburban area of Hanoi. BMH has its own infectious disease facility, the Center for Tropical Diseases (CTD), separate from NHTD (Figure 1).

In early January 2020, BMH established 2 dedicated COVID-19 screening triage clinics for suspected cases. These clinics were located in separate areas from other departments of the hospital: the first was next to the main gate, and the second was set up near the CTD (Figure 1). Healthcare workers from the CTD operated both clinics, 1 doctor and 2 nurses working per shift. All patients were required to be screened at the clinics before they received any other services. Clinic staff performed general clinical examinations, gathered epidemiologic data, and classified whether each patient had a suspected case using general criteria issued by the Ministry of Health (MoH), including having >1 suspicious symptom (fever, cough, shortness of breath) and having a history of traveling through epidemic areas or having close contact with a patient with confirmed COVID-19 during the preceding 14 days. Before March 12, nasopharyngeal swab specimens from the suspected cases were transferred to the NIHE for SARS-CoV-2 confirmation. Beginning March 13, the specimens were processed and confirmed at BMH itself. Patients with suspected cases were transferred immediately, in dedicated vehicles, to the second branch of NHTD, even if test results had not yet been received. 

### Study Design and Data Collection

We conducted a desk review of available documents, patient records, and public data collected during March 17–April 15, 2020. We retrieved demographic data from the official COVID-19 database provided by General Department of Preventive Medicine (https://ncov.vncdc.gov.vn). Symptoms and treatment data were systematically collected from the official MoH COVID-19 database (https://ncov.moh.gov.vn) and the MoH official press release website (https://suckhoedoisong.vn).

### Quarantine Measures

We established different definitions of suspected cases, as well as a hierarchy of contacts, between the BMH outbreak and standards management in Vietnam in general. The MoH’s general guidelines defined a suspected case as illness in a person who had >1 suspicious symptom and had epidemiologic criteria such as travel abroad or direct contact with suspected cases. Patients with suspected cases were put in centralized quarantine for 14 days and tested for SARS-CoV-2. The contacts were categorized at 3 levels: F1 for close contacts of persons with laboratory-confirmed COVID-19 cases, F2 for close contacts of F1 persons, and F3 for close contacts of F2 persons. F1 persons were also placed in centralized quarantine and tested, whereas F2 and F3 persons were isolated and monitored at home. When a community had several confirmed cases and the index patients had multiple complicated contacts, the lockdown of a small administrative unit (usually at the commune level) was carried out.

In the outbreak at BMH, all persons who visited the hospital during March 10–March 20 were considered as the F1 group, regardless of their exposure to laboratory-confirmed cases. For contact tracing, 4 levels of contacts were followed up, from F1 to F4 (F4 comprised close contacts of F3), which is one level higher than the general guideline. F1 and F2 persons were quarantined at a centralized area, and F3 and F4 persons isolated at home (Table). Affected departments at the BMH area were isolated as soon as cases were detected, and lockdown of the entire hospital was implemented after the confirmation of 8 cases and 4 affected departments (Figure 1).

**Table:** Definitions, risk assessment, mitigation strategy, and numbers of contacts traced in the COVID-19 outbreak at Bach Mai Hospital, Hanoi, Vietnam, March–April, 2020

Group	Definition	Risk assessment	Strategy	No. tracings
F1	All patients who visited the hospital, including discharged, transferred out, and outpatients	Highest	Quarantined at centralized centers for 14 d	27,893
	Family caregivers of patients		Test for SARS-CoV-2	
	Healthcare workers		Daily health assessment by healthcare workers	
	Medical students and visiting scholars			
	People who visited patients			
	People from catering company, including nonlocal staff			
	Private hired caregivers for patients			
F2	Close contacts of F1	High		
F3	Close contacts of F2	Medium	Isolated at home for 14 d	24,346
F4	Close contacts of F3	Low	Self-monitoring for at-risk symptoms	
			Remote health monitoring by local healthcare workers	

### SARS-COV-2 Testing Strategy

We tested all F1 persons quarantined at BMH for SARS-CoV-2 using RT-PCR in the microbiology department; persons at the CTD, NHI, and neurology department (ND) were tested 3 times to confirm the situations in these departments, and all others at BMH were tested once. Because of the requirement of >2 negative tests before a person was released from quarantine, Hanoi Center for Disease Control conducted an additional retest for all confirmed cases before the removal of lockdown. The test kits were either donated by the WHO or provided by Viet-A Corporation. In total, an estimated 15,000 tests were analyzed for quarantined persons at BMH. Each test cost »$30 USD.

F1 and F2 persons who were traced and quarantined in the community were provided >1 test by the local Center for Disease Control. The numbers of tests per person depended on the decision of the local steering committee, which considered the occurrence of symptoms and laboratory capacity.

The study was approved by the BMH ethics committee. We applied all ethics considerations needed according to MoH or by its designees.

## Results

### Timeline and Outbreak Management at BMH

**Figure 2:**
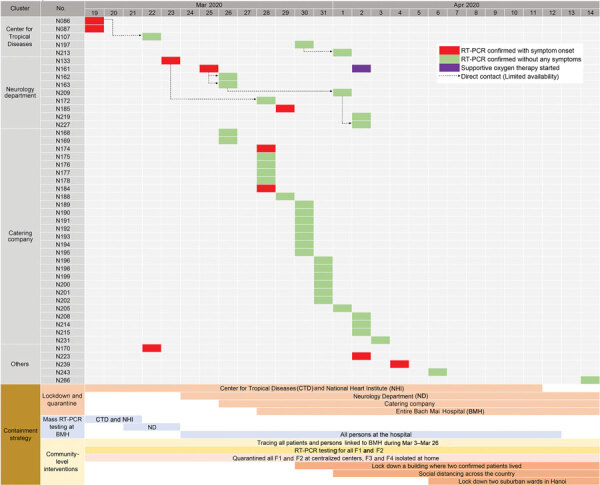
Details of severe acute respiratory syndrome coronavirus 2 infections positive cases and timeline of containment strategy for infections related to Bach Mai Hospital, Hanoi, Vietnam. RT-PCR, reverse transcription PCR.

During March 18–April 14, a total of 46 laboratory-confirmed COVID-19 cases were detected at BMH. The mean age of the patients was 44.9 years, and 80.4% were female. Ten (21.7%) patients were symptomatic; 91.3% had a history of admission to BMH or working or visiting an institute in the BMH complex, including healthcare workers (4.4%), nonclinical staff (58.7%), patients (13.0%), and family caregivers (15.2%) (Figure 2).

Case 86 was in a female nurse working at the HIV outpatient clinic of the CTD. On March 11, she had chest tightness and pain and was admitted to the NHI; her diagnosis was a clinical manifestation of preexisting hypertension illness. She had multiple contacts with CTD staff during lunch and break periods, including the patient with case 87, a 33-year-old female nurse working at the COVID-19 screening clinic who developed fever (38.5°C) and dry cough on March 18 and had a positive test for SARS-CoV-2 on the same day (Figure 2). Quarantine and mass testing were imposed for all of CTD on March 19, involving 159 healthcare workers. NHI was also put in quarantine on the same day; this quarantine included 84 persons (Figure 1).

Case 133 was in a 66-year-old woman who was admitted to Lai Chau General Hospital for stroke on February 29 and was transferred to the BMH neurology department (Figure 2). On March 22, she was transferred back to Lai Chau General Hospital because she developed fever and cough and tested positive for SARS-CoV-2. Quarantine was imposed at the neurology department on March 24 for a total of 252 persons: 162 healthcare workers, 36 patients, and 54 caregivers (Figure 1).

Of the 46 confirmed cases, 27 were from the hospital catering company (Figure 1). These persons provided food and drinks for staff and patients in the hospital and managed the hospital canteens and cleaning tasks. Thus, they moved throughout the hospital and worked close to one another. The reason for the transmission among the company staff might have been the close contact they had during their work without adequate protective equipment. Of the 91 catering company staff who worked at BMH, 28% were SARS-CoV-2 positive. Cases 174 and 184 were symptomatic, with fever and cough, but the others were asymptomatic.

On March 28, the quarantine was extended to all of BMH. A total of 7,664 persons were quarantined: 6,258 healthcare workers and other staff members, 793 inpatients, and 613 of the patients’ related family caregivers (Figure 1).

BMH stopped new admissions on March 20, except for patients with severe and critical conditions. A total of 5,113 inpatients were transferred to local provincial hospitals or other specialized hospitals in Hanoi. These patients had non–COVID-19-related illnesses and were considered safe to transfer; they were managed as F1 persons and received preventive measures from the local government and Center for Disease Control. A total of 793 patients with non–COVID-19-related illnesses required treatment at BMH because of the severity of their illness. These patients were managed with a high level of infection control, including spacing beds >2 m apart, ensuring that all healthcare workers used personal protective equipment, and having healthcare workers using N95 masks when performing aerosol-generating procedures associated with viral spread. In addition, only 1 healthcare worker at a time was allowed contact with a patient, except when a medical intervention required >1 person. Family caregivers were not permitted to have direct contact with patients. Body temperature measurement and mandatory medical reporting for all persons in and out of the hospital, enhanced room air flow, and retraining in infection prevention and control (IPC) were implemented for all the staff. Separate entryways for new emergency cases and routine dialysis patients were set. Other routine outpatients, such as patients with diabetes, hepatitis, or cardiovascular disease, were asked to delay their regular visits and go to local hospitals for care and treatment.

### Contact Tracing and Outbreak Containment in the Community

F1 persons were categorized into 7 groups. Four groups had registered information at BMH: healthcare workers, visiting scholars and students, nonclinical staff, and patients (both inpatients and outpatients). The other 3 groups, family, hired caregivers, and other persons who visited patients, could be found only by epidemiologic investigation, self-reported or reported in the local community. In addition, information on all cases was widely available on social media and media outlets, alerting members of the general community about potential exposure if they were at the hospital.

Healthcare workers, visiting scholars and students, severely or critically ill inpatients, and their family and hired caregivers were quarantined and managed at BMH. All other BMH cases from March 10–20 (including 5,113 transferred patients) were traced and managed by the local Hanoi steering committee. A total of 52,239 persons were followed up in the community. Among those who were traced, 27,893 F1 and F2 persons were put in quarantine (Table). Nearly 30,000 RT-PCR tests were performed on F1 and F2 persons in the community.

## Discussion

We describe how nosocomial transmission in a large hospital was contained through extensive testing of all possibly exposed persons, even those without any symptoms; whole hospital quarantine for >2 weeks; contact tracing in the community; and quarantine of all contacts. From the beginning of the COVID-19 pandemic, testing strategy has been an essential intervention in preventing community spread of COVID-19 in Vietnam. As of May 13, 2020, >275,000 SARS-CoV-2 RNA tests had been conducted in Vietnam; the proportion of tests per confirmed case was »950 (*12*). These measures were put in place to prevent a generalized epidemic and a heavy burden on the healthcare system, which generally was overloaded, with only »9 doctors and 15 nurses per 10,000 population (*13*). One advantage during the outbreak at BMH was the laboratory capacity and available RT-PCR test kits provided by a local company. About 15,000 tests were done for persons quarantined at the hospital. More than half of these tests were analyzed at BMH itself, which helped to greatly reduce the waiting time for detecting cases. In addition, by March 21, there were 22 licensed laboratories, including 6 provincial Center for Disease Controls, able to perform RT-PCR tests for SARS-CoV-2 across the country, which increased the local case detection capacity.

All of BMH was quarantined after 8 cases of COVID-19 were detected in 4 departments. All persons linked to the hospital, including healthcare workers, inpatients, outpatients, visitors, and close contacts of these persons within 14 days before the lockdown (27,893 persons), were considered as having suspected cases, placed in centralized quarantine, and tested. Modeling suggested that active case tracing and early testing had a major effect on reducing the community transmission of COVID-19, up to 80% (*14*), and the outcomes from outbreak containment at BMH could provide good empirical evidence for this result. Active case tracing has been implemented in several other countries and has shown remarkable effectiveness (*15*–*18*).

Quarantine for all the contacts was the major factor for successful outbreak containment at BMH. However, quarantine was not always an acceptable solution for many other settings because of the lack of resources, facilities, or policy support (*19*,*20*). In the case of BMH, the decision on the whole-hospital quarantine was made by considering multiple criteria. The first advantage was the hospital’s beds for transferred-out patients and the new 9-story building that could be used for the accommodation of quarantined persons. Second, the hospital contingency fund and support from the Hanoi city council were rapidly mobilized for food, drinks, and other necessities. In addition, the transmission from an unknown index case might have been the tip of an iceberg of undetected of community transmission in Hanoi that encouraged aggressive actions to prevent widespread community transmission.

Only 10 symptomatic cases were found among the 46 laboratory-confirmed COVID-19 cases in the outbreak (21.7%); 1 patient needed intensive care with mechanical ventilation (2.2%), and there were no deaths. Several large investigations with a similar approach to active case tracking efforts also showed a high rate of asymptomatic patients among persons who tested positive for SARS-CoV-2. For example, in a cohort of 829 employees who worked at Rutgers University and associated hospitals in New Jersey, USA, the prevalence of asymptomatic SARS-CoV-2 cases was 65.9% (E.S. Barrett et al., unpub. data, https://doi.org/10.1101/2020.04.20.20072470). A large population screening in Iceland showed that the positive rate among 13,080 nontargeted citizens was 0.8%, and 43% of SARS-CoV-2 positive cases were asymptomatic (*21*). In a homeless shelter in Boston, the asymptomatic rate among persons who tested positive for SARS-CoV-2 was 87.8% (*22*). These results emphasize the importance of detecting mild or asymptomatic COVID-19 cases because they may be vectors for transmission (*23*; D.C. Buitrago-Garcia et al., unpub. data, https://doi.org/10.1101/2020.04.25.20079103). 

The relatively low rate of transmission to healthcare workers might be the result of use of personal protective equipment and masks, as well as other IPC activities. In addition, the outbreak occurred during a generally cool time of year, so opening windows and doors was still practiced in most of areas of the hospital, which could help to lower transmission risk, compared with having to use air conditioning during the warmer season (*24*). Most of the cases were in nonclinical staff members, which might be the result of high frequency of exposure with lack of protective equipment as well as work in crowded conditions in the kitchen and canteen. After the first cluster was detected at the CTD, a higher level of infection control was implemented, including retraining in IPC measures for all staff. However, the compliance of nonmedical staff was inadequate, which might be the result of a lack of adequate information, training, and personal protective equipment, as well as management possibly underestimating the severity of the situation. This finding illustrates the importance for healthcare facilities to protect their nonclinical staff by providing appropriate training and adequate protective equipment, because these staff members may be both victims and vectors to other staff and patients (*25*,*26*).

The outbreak at BMH contributed to a decision to implement a social distancing campaign throughout Vietnam during April 1–April 14 and in Hanoi for an additional week after that. We found that the BMH outbreak uncovered both nosocomial and unexplained cases likely to have resulted from community transmission. The social distancing campaign might have contributed to reducing community transmission, as indicated in several other settings (*27*,*28*). In addition, experiences from the containment of the SARS outbreak in 2003 (*29**,**30*), which also occurred at BMH, helped hospital management make quarantine decisions faster. Many frontline healthcare workers who were present during the SARS outbreak 2003 were still working at BMH and contributed to the management, planning, and processing of the COVID-19 outbreak containment. Although containment in the BMH COVID-19 outbreak was successful, the index case was not found.

Our investigation is subject to several limitations. First, we could not estimate the coverage of contact tracing in the community because of the lack of information for some at-risk groups that did not register in the database (family/private caregivers and persons who visited patients). Second, because we did not interview all the patients who tested positive, the source and index cases were not fully interpreted. Finally, we did not perform a complete outbreak investigation, which reduced the validity of the containment outcomes and made it difficult to compare this study with other studies.

In conclusion, the COVID-19 outbreak containment at BMH is a noteworthy example in which a major university hospital was quarantined to prevent further community transmission. Containment of the outbreak in BMH could serve as an example for other settings that are experiencing new outbreaks of this highly transmissible disease. 

We suggest several recommendations to prevent hospital COVID-19 nosocomial outbreaks. Strict triage stations should be established at all entrances; healthcare workers, nonclinical staff, and contract workers should be monitored and those with symptoms recommended to stay home if ill; and other key IPC measures should be instituted at the hospital according to the hierarchy of IPC controls. Protective equipment should be provided to all staff, both clinical and nonclinical, as well as training in how to use it correctly. In addition, cases of severe viral pneumonia should be monitored closely, with SARS-CoV-2 testing recommended when all other possible causes have been excluded. High-risk groups, such as patients with severe acute respiratory infections, healthcare workers, and elderly patients, should also be strictly monitored.
